# Draft Genome Sequence of Aeromonas caviae Strain L12, a Quorum-Sensing Strain Isolated from a Freshwater Lake in Malaysia

**DOI:** 10.1128/genomeA.00079-15

**Published:** 2015-03-05

**Authors:** Kok-Gan Chan, Pui-San Chin, Kok Keng Tee, Chien-Yi Chang, Wai-Fong Yin, Kit-Yeng Sheng

**Affiliations:** a Division of Genetics and Molecular Biology, Institute of Biological Sciences, Faculty of Science, University of Malaya, Kuala Lumpur, Malaysia; b Centre of Excellence for Research in AIDS (CERiA), Department of Medicine, Faculty of Medicine, University of Malaya, Kuala Lumpur, Malaysia; c Interdisciplinary Computing and Complex BioSystems (ICOS) research group, School of Computing Science, Claremont Tower, Newcastle University, Newcastle upon Tyne, United Kingdom; d The Centre for Bacterial Cell Biology, Medical School, Newcastle University, Richardson Road, Newcastle upon Tyne, United Kingdom

## Abstract

Here, we present the draft genome sequence of Aeromonas caviae strain L12, which shows quorum-sensing activity. The availability of this genome sequence is important to the research of the quorum-sensing regulatory system in this isolate.

## GENOME ANNOUNCEMENT

Aeromonads are Gram-negative, non-spore-forming, facultative anaerobic, rod-shaped bacteria. Members of Aeromonas can be found in aquatic environments. Aeromonas caviae is a motile mesophilic species in the Aeromonas genus, which is often associated with human infections (1). A. caviae has been isolated from patients with sepsis and wounds, gastroenteritis, and systemic infections (2–4). A process known as quorum sensing was described to be responsible for the regulation of virulence factors (5–8). In this work, A. caviae strain L12 was isolated from a freshwater lake in Malaysia and subjected to whole-genome sequencing.

Genomic DNA of A. caviae strain L12 was extracted using a MasterPure DNA purification kit (Epicentre, Madison, WI, USA) according to the manufacturer’s recommended protocol. The quality of the purified genomic DNA was determined using a NanoDrop spectrophotometer (Thermo Scientific) and Qubit 2.0 fluorometer (Life Technologies) (9). Whole-genome sequencing was done using a paired-end sequencing method with the HiSeq 2500 platform (Illumina, Inc., San Diego, CA, USA) (10). The sequences were trimmed with a quality threshold of Q30 and *de novo* assembly was done using CLC Genomics Workbench version 7.0 (CLC Bio, Aarhus, Denmark) (11). The quality trimming yielded a total count of 4,908,771 paired-end reads with an average read length of 80.8 bp. The draft genome size of A. caviae strain L12 is 4,376,717 bp with 91 contigs with an average coverage of 100.6-fold. The mean contig read length is 126,255-bp and the maximum contig read length is 294,904 bp. Its G+C content is 61.7%, consisting of 30.7% cytosine and 31.0% guanine. Gene prediction and annotation was done using PROKKA version 1.10 resulting in 3,958 open reading frames (ORFs) and 4,089 genes (12). The number of RNAs was also predicted using PROKKA version 1.10. There were 3 rRNAs and 84 tRNAs identified.

Genes that were annotated were then searched against the NCBI database using BLAST (13) to confirm their identity and searched against the UniProt database (14) to further understand their functions and characteristics. From the annotated genes of the draft genome of strain L12, several putative quorum-sensing related genes were found, such as transcriptional activator protein LuxR, homoserine and homoserine lactone efflux proteins, regulatory protein LuxO, and autoinducer 2 sensor kinase/phosphatase LuxQ. It is hoped that this genome sequence will provide a better understanding of quorum-sensing regulation in this isolate.

### Nucleotide sequence accession numbers.

This whole-genome shotgun project has been deposited at DDBJ/EMBL/GenBank under accession no. JWJP00000000. The version described in this paper is version JWJP01000000.
